# Nocardia niwae Disseminated Nocardiosis: A Novel Species Presenting Concurrently With Lung Adenocarcinoma

**DOI:** 10.7759/cureus.31246

**Published:** 2022-11-08

**Authors:** Natasha Emanuel, Nikhut Siddique, John Greene, Yanina Pasikhova, Austin Morrison, Mark Ledbetter, Guy Handley

**Affiliations:** 1 Clinical Pharmacy, Moffitt Cancer Center, Tampa, USA; 2 Internal Medicine, University of South Florida Morsani College of Medicine, Tampa, USA; 3 Internal Medicine, Moffitt Cancer Center, Tampa, USA

**Keywords:** gram-positive bacillus, immunocompromised hosts, lung adenocarcinoma, disseminated nocardiosis, nocardia niwae

## Abstract

Nocardia includes over 90 species of filamentous gram-positive bacilli that may cause disease in immunocompromised or immunocompetent hosts. Presentations may include pulmonary, 4, cutaneous, or disseminated infections. Tissue diagnosis may be required as it may mimic alternative etiologies. There is a paucity of data regarding rarer species of Nocardia. Intraspecies variability in antimicrobial susceptibility limits many treatment regimens to in-vitro activity data and treatment regimens often must be tailored to individual patients based on microbiologic cultures. We describe the case of a 63-year-old female who presented with disseminated *Nocardia niwae*, a species that was previously first identified in Florida for which little clinical data is known, along with concurrent lung adenocarcinoma with pulmonary and central nervous system lesions. Typical susceptibility patterns are discussed along with potential side effects of antimicrobial therapy.

## Introduction

Nocardia infections cause about 500-1,000 new cases annually in the United States [1]. Nocardia is a filamentous gram-positive branching bacillus part of the Actinomycetes group. It is a ubiquitous environmental organism found in soil, decomposing vegetation, fresh water, and saltwater [2]. Transmission occurs through inhalation or direct inoculation, and the disease can manifest as pulmonary, central nervous system (CNS), cutaneous, or disseminated infection [1]. The one-year mortality can be up to 66% [3]. Roughly two-thirds of patients who present with Nocardia infections are immunocompromised [1]. There are over 90 recognized species; 50 of these are pathogenic in humans [1]. More common species include *N. abscessus*, *N. farcinica* complex, *N. brasiliensis*, *N. asteroids*, *N. nova* complex, and *N. cyriacigeorgica* [1,4]. There is limited data regarding rarer species, and antimicrobial susceptibility varies greatly between species. Much of the literature regarding treatment regimens or susceptibility profiles exist as in-vitro activity data. Resistance mechanisms are common, leading to difficulty in choosing empiric antimicrobial regimens. Many species have typically shown to be susceptible to sulfamethoxazole/trimethoprim, linezolid, and amikacin [4]. Empiric therapy for Nocardia infections is generally recommended to include at least a two-drug regimen with an additional third agent for more severe or disseminated infections [2]. We describe a 63-year-old female who presented with disseminated *Nocardia niwae*.

## Case presentation

**Figure 1:**
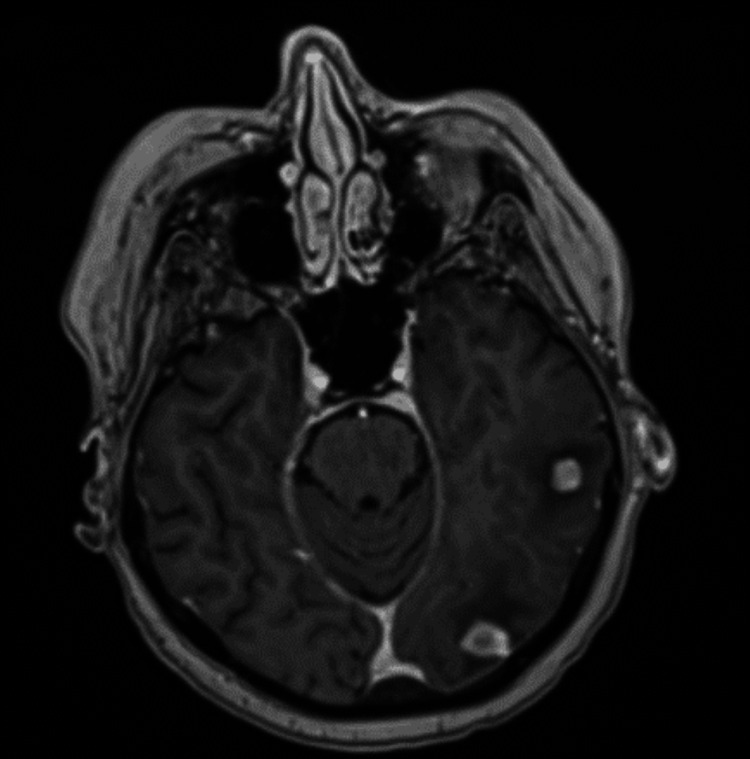
Post-contrast axial T1-weighted magnetization-prepared rapid gradient-echo (MPRAGE) magnetic resonance imaging (MRI) demonstrating a sample of left-sided intracerebral lesions

**Figure 2:**
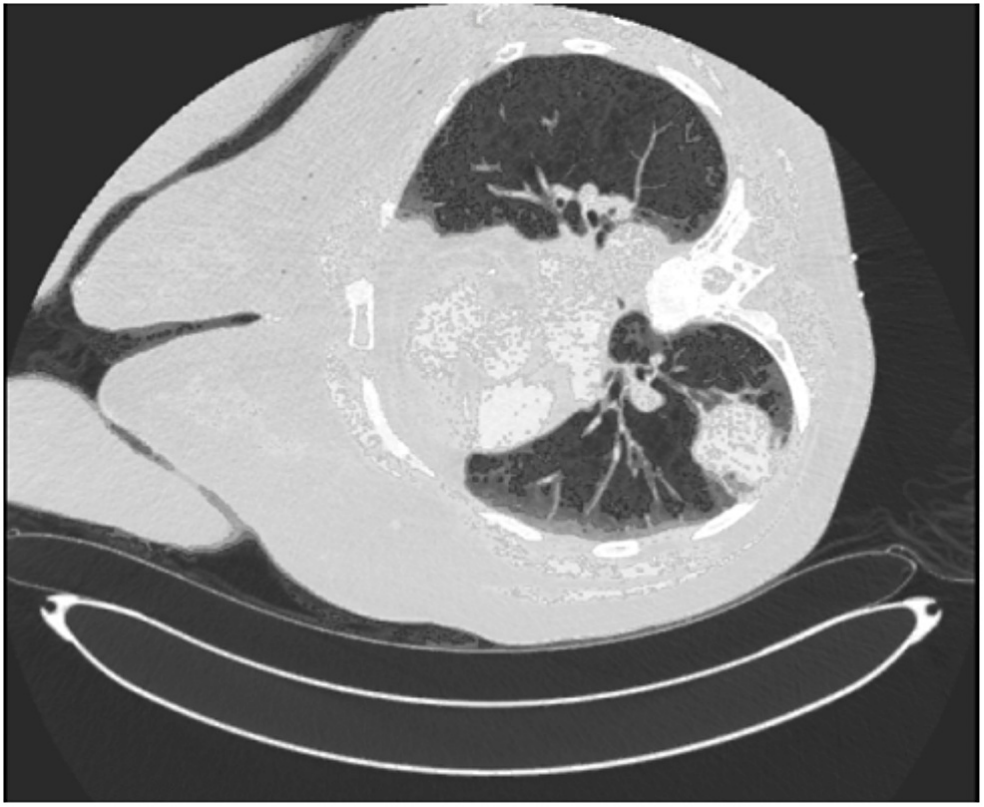
Positron emission tomography (PET) scan demonstrating right nodular lung lesion

A 63-year-old female with no significant past medical history other than significant cigarette smoking history, presented with progressive complaints of word-finding difficulty, headaches, gait instability requiring the use of a walker, and visual disturbance over several weeks. She underwent brain magnetic resonance imaging (MRI) demonstrating multiple discrete solid nodular and ring-enhancing lesions up to 1.1 cm throughout the supratentorial and infratentorial space concerning for metastatic malignancy. Further workup with computerized tomography (CT) of the lung revealed a 0.9 cm solitary nodule in the superior segment of the left lung concerning for primary lung malignancy. A biopsy specimen of the lung lesion was obtained and thought to be consistent with lung adenocarcinoma. She was initiated on dexamethasone 4 mg orally every 12 hours with a 6-week taper course and underwent Whole Brain Radiation Therapy (WBRT) with hippocampal avoidance with 3000 cGy dose in 10 fractions. There was minimal improvement in symptoms for which she was referred to our institution for a second opinion. There was interval enlargement in most of the brain lesions with the new development of metabolically active lung nodular lesions on positron emission tomography (PET) scan. A representative image of brain and lung lesions is provided in Figures 1-2. A second lung nodule biopsy was performed. Pathology demonstrated necrotic debris and granulomatous inflammation. The tissue culture was significant for filamentous-branching gram-positive rods with modified acid-fast bacilli (AFB) stain positive for Nocardia species. The patient was admitted and Infectious Diseases was consulted. Given the brain lesions, there was a concern for CNS involvement of disseminated nocardiosis. The patient was recommended to undergo a brain biopsy for definitive diagnosis due to a differential diagnosis that included malignancy, tuberculosis, or primary lymphoma among others; however, the procedure was felt to be too high-risk. She was subsequently empirically treated for disseminated nocardiosis with CNS involvement with sulfamethoxazole/trimethoprim 800 mg/160 mg two tablets every 8 hours, imipenem/cilastatin 1 g IV every 8 hours, and amikacin 15 mg/kg IV every 24 hours. Amikacin was subsequently changed to linezolid 600 mg orally every 12 hours to achieve increased CNS penetration. Due to social factors, imipenem/cilastatin was changed to ceftriaxone 2 g IV twice daily for ease of administration at home. There was a concern for suboptimal activity with this particular change and therefore, moxifloxacin was added while awaiting organism identification and susceptibility testing. Final discharge antibiotics were ceftriaxone 2 g IV twice daily, sulfamethoxazole/trimethoprim 800/160 2.5 tablets three times per day, linezolid 600 mg orally twice daily, and moxifloxacin 400 mg orally daily. Two weeks after the initiation of antimicrobials the patient developed hyponatremia with nausea, vomiting, and falls thought to be secondary to the higher dosage of sulfamethoxazole/trimethoprim.

Final microbiological cultures identified N. niwae as susceptible to ceftriaxone, linezolid, minocycline, sulfamethoxazole/trimethoprim, tobramycin, amikacin, and clarithromycin. It was resistant to ciprofloxacin and had intermediate susceptibility to doxycycline (Table 1). The patient’s antibiotics were adjusted to ceftriaxone 2 g IV every 12 hours and minocycline 200 mg orally every 12 hours based on the susceptibility patterns and patient-specific factors. At a 6-week follow-up visit, the brain MRI did not show improvement in any lesions, and the CT chest demonstrated the development of new nodules and growth of known lesions. Her ceftriaxone was changed to sulfamethoxazole/trimethoprim 800/160 two tablets three times daily out of concern of antimicrobial failure and a repeat biopsy was ordered. A biopsy of a separate nodule in the right lung revealed lung adenocarcinoma. Her functional status progressively declined, and she was admitted with an acute renal injury and a metastatic pleural effusion. Ultimately due to progressive decline, the patient was unable to start anti-neoplastic therapy and elected to transition to hospice care.

**Table 1:** In-vitro antimicrobial susceptibility testing for Nocardia niwae

Antibiotic	MIC (mcg/ml)	Susceptibility
Amikacin	≤1	Susceptible
Amoxicillin/Clavulanate	32	Resistant
Ceftriaxone	≤4	Susceptible
Ciprofloxacin	4	Resistant
Clarithromycin	0.25	Susceptible
Doxycycline	2	Intermediate
Linezolid	≤1	Susceptible
Minocycline	≤1	Susceptible
Tobramycin	≤1	Susceptible
Trimethoprim/Sulfamethoxazole	4	Susceptible

## Discussion

Most literature surrounding Nocardia infections are observational studies and case reports. In immunosuppressed patients, pulmonary nocardiosis is the most common clinical presentation [5]. Patients may present with abscesses/cavities, pulmonary nodules, or lobar infiltrates. Disseminated Nocardia occurs in an estimated 1/3 of patients. It is most often from a pulmonary source, however, frequently involves the CNS, other internal organs, and the skin. Dissemination is most often observed in immunocompromised patients and should be ruled out when Nocardia is isolated [5].

*N. niwae* has only been identified within the United States and was first identified in Florida where four out of the only five reported cases have occurred [6]. Moser and colleagues first identified this species of Nocardia through taxonomic analysis of a lung biopsy in 2011 using 16s rRNA gene sequence analysis. Four patients are presented with clinical data limited to the tissue tested for the organism [6]. Datta and colleagues published a case report on a patient with N. niwae disseminated infection [7]. The 86-year-old patient had lung lesions and an axillary lesion presumed to be metastatic squamous cell cancer. He completed chemoradiotherapy, immunotherapy, and steroids for presumed immunotherapy-related pneumonitis. He was treated for several weeks with antimicrobials for a possible right elbow synovial infection before repeat aspiration isolated *N. niwae*. Treatment was initiated with sulfamethoxazole/trimethoprim and meropenem and was also complicated by hyponatremia due to sulfamethoxazole/trimethoprim [7]. The patient expired and the autopsy showed *N. niwae*, but no evidence of lung carcinoma.

Similarly, in our patient, the lung lesions and brain lesions were initially thought to be malignant, however, subsequent biopsy cultures isolated Nocardia, and treatment was initiated based on available literature regarding typical susceptibility patterns. Several published case reports note disseminated Nocardia occurring in patients with and without a malignancy and may mimic metastases [8,9]. Our case demonstrates the critical importance of tissue biopsy for definitive diagnosis, particularly when multiple diseases may be present. Prompt diagnosis and treatment for nocardiosis are crucial to successful outcomes.

Nocardia susceptibility to antimicrobials is highly dependent upon the species and site of infection. Considering the pharmacokinetic and pharmacodynamic factors of each antimicrobial option, sulfamethoxazole/trimethoprim, imipenem, and amikacin were chosen for the initial treatment. Sulfamethoxazole/trimethoprim has been proven to be a mainstay of treatment for Nocardia infections based on its high susceptibility profile for many species [5]. Severe hyponatremia may occur with sulfamethoxazole/trimethoprim and is often dose-related. Patients who receive treatment with a trimethoprim component that is more than 8 mg/kg per day for three days or longer, are more likely to experience hyponatremia [10]. Our patient was receiving approximately 12.5 mg/kg daily trimethoprim component and experienced profound hyponatremia leading to the discontinuation of the medication. Imipenem, amikacin, and linezolid have also shown high in-vitro activity against many Nocardia species.

Our case is the second complete clinical report of a rare species of Nocardia that is geographically limited primarily to the state of Florida. In addition, our case demonstrates the critical importance of tissue diagnosis when multiple etiologies are considered, provides information for other providers with future cases, and informs of potential side effects, such as hyponatremia, encountered with traditional antimicrobials for this disease.

## Conclusions

This case report highlights the rare species of *N. niwae* presenting as disseminated nocardiosis mimicking metastatic malignancy. Disseminated nocardiosis should always be in the differential in patients who present with pulmonary nodules and enhancing brain lesions, especially in immunocompromised hosts. Definitive diagnosis with biopsy followed by prompt initiation of empiric antibiotic therapy should be considered to avoid misdiagnosis of nocardiosis and is critical for treatment success. Close follow-up and monitoring should be established to ensure concomitant diagnoses, such as malignancy, are also not present. Susceptibility testing should then guide final treatment regimens.

## References

[1] Hamdi AM, Fida M, Deml SM, Abu Saleh OM, Wengenack NL (2020). Retrospective analysis of antimicrobial susceptibility profiles of Nocardia species from a tertiary hospital and reference laboratory, 2011 to 2017. Antimicrob Agents Chemother.

[2] Wilson JW (2012). Nocardiosis: updates and clinical overview. Mayo Clin Proc.

[3] Davidson N, Grigg MJ, Mcguinness SL, Baird RJ, Anstey NM (2020). Safety and outcomes of linezolid use for nocardiosis. Open Forum Infect Dis.

[4] Wang HL, Seo YH, LaSala PR, Tarrand JJ, Han XY (2014). Nocardiosis in 132 patients with cancer: microbiological and clinical analyses. Am J Clin Pathol.

[5] Ambrosioni J, Lew D, Garbino J (2010). Nocardiosis: updated clinical review and experience at a tertiary center. Infection.

[6] Moser BD, Klenk HP, Schumann P (2011). Nocardia niwae sp. nov., isolated from human pulmonary sources. Int J Syst Evol Microbiol.

[7] Datta R, Kramer E, Reinhart H, Campbell S, Wong E, Gupta S (2018). Menace elbow: disseminated nocardiosis. Am J Med.

[8] Karan M, Vučković N, Vuleković P, Rotim A, Lasica N, Rasulić L (2019). Nocardial brain abscess mimicking lung cancer metastasis in immunocompetent patient with pulmonary nocardiasis: a case report. Acta Clin Croat.

[9] Arjun R, Padmanabhan A, Reddy Attunuru BP, Gupta P (2016). Disseminated nocardiosis masquerading as metastatic malignancy. Lung India.

[10] Tsapepas D, Chiles M, Babayev R, Rao MK, Jaitly M, Salerno D, Mohan S (2016). Incidence of hyponatremia with high-dose trimethoprim-sulfamethoxazole exposure. Am J Med.

